# Conjugated Linoleic Acid Administration Induces Amnesia in Male Sprague Dawley Rats and Exacerbates Recovery from Functional Deficits Induced by a Controlled Cortical Impact Injury

**DOI:** 10.1371/journal.pone.0169494

**Published:** 2017-01-26

**Authors:** Rastafa I. Geddes, Kentaro Hayashi, Quinn Bongers, Marlyse Wehber, Icelle M. Anderson, Alex D. Jansen, Chase Nier, Emily Fares, Gabrielle Farquhar, Amita Kapoor, Toni E. Ziegler, Sivan VadakkadathMeethal, Ian M. Bird, Craig S. Atwood

**Affiliations:** 1 Division of Geriatrics and Gerontology, Department of Medicine, University of Wisconsin-Madison School of Medicine and Public Health, Wisconsin, United States of America; 2 Assay Services Unit and Institute for Clinical and Translational Research Core Laboratory, National Primate Research Center, University of Wisconsin-Madison, Wisconsin, United States of America; 3 Department of Obstetrics and Gynecology, University of Wisconsin-Madison School of Medicine and Public Health, Wisconsin, United States of America; 4 Geriatric Research, Education and Clinical Center, Veterans Administration Hospital, Madison, Wisconsin, United States of America; 5 School of Exercise, Biomedical and Health Sciences, Edith Cowan University, Joondalup, Western Australia, Australia; University of Toronto, CANADA

## Abstract

Long-chain polyunsaturated fatty acids like conjugated linoleic acids (CLA) are required for normal neural development and cognitive function and have been ascribed various beneficial functions. Recently, oral CLA also has been shown to increase testosterone (T) biosynthesis, which is known to diminish traumatic brain injury (TBI)-induced neuropathology and reduce deficits induced by stroke in adult rats. To test the impact of CLA on cognitive recovery following a TBI, 5–6 month old male Sprague Dawley rats received a focal injury (craniectomy + controlled cortical impact (CCI; n = 17)) or Sham injury (craniectomy alone; n = 12) and were injected with 25 mg/kg body weight of Clarinol^®^ G-80 (80% CLA in safflower oil; n = 16) or saline (n = 13) every 48 h for 4 weeks. Sham surgery decreased baseline plasma progesterone (P_4_) by 64.2% (from 9.5 ± 3.4 ng/mL to 3.4 ± 0.5 ng/mL; p = 0.068), T by 74.6% (from 5.9 ± 1.2 ng/mL to 1.5 ± 0.3 ng/mL; p < 0.05), 11-deoxycorticosterone (11-DOC) by 37.5% (from 289.3 ± 42.0 ng/mL to 180.7 ± 3.3 ng/mL), and corticosterone by 50.8% (from 195.1 ± 22.4 ng/mL to 95.9 ± 2.2 ng/mL), by post-surgery day 1. CCI injury induced similar declines in P_4_, T, 11-DOC and corticosterone (58.9%, 74.6%, 39.4% and 24.6%, respectively) by post-surgery day 1. These results suggest that both Sham surgery and CCI injury induce hypogonadism and hypoadrenalism in adult male rats. CLA treatment did not reverse hypogonadism in Sham (P_4_: 2.5 ± 1.0 ng/mL; T: 0.9 ± 0.2 ng/mL) or CCI-injured (P_4_: 2.2 ± 0.9 ng/mL; T: 1.0 ± 0.2 ng/mL, p > 0.05) animals by post-injury day 29, but rapidly reversed by post-injury day 1 the hypoadrenalism in Sham (11-DOC: 372.6 ± 36.6 ng/mL; corticosterone: 202.6 ± 15.6 ng/mL) and CCI-injured (11-DOC: 384.2 ± 101.3 ng/mL; corticosterone: 234.6 ± 43.8 ng/mL) animals. In Sham surgery animals, CLA did not alter body weight, but did markedly increase latency to find the hidden Morris Water Maze platform (40.3 ± 13.0 s) compared to saline treated Sham animals (8.8 ± 1.7 s). In CCI injured animals, CLA did not alter CCI-induced body weight loss, CCI-induced cystic infarct size, or deficits in rotarod performance. However, like Sham animals, CLA injections exacerbated the latency of CCI-injured rats to find the hidden MWM platform (66.8 ± 10.6 s) compared to CCI-injured rats treated with saline (30.7 ± 5.5 s, p < 0.05). These results indicate that chronic treatment of CLA at a dose of 25 mg/kg body weight in adult male rats over 1-month 1) does not reverse craniectomy- and craniectomy + CCI-induced hypogonadism, but does reverse craniectomy- and craniectomy + CCI-induced hypoadrenalism, 2) is detrimental to medium- and long-term spatial learning and memory in craniectomized uninjured rats, 3) limits cognitive recovery following a moderate-severe CCI injury, and 4) does not alter body weight.

## Introduction

Traumatic brain injury (TBI) is a major public health problem [1] due to the relatively high incidence rate (106 per 100,000 globally, [2]) and the lack of effective treatments. TBI-related visits to the emergency department (ED), hospitalizations, and deaths are higher in males than in females, while the elderly are at a significantly higher risk of TBI-related hospitalizations, regardless of gender [2]. The failure of clinical trials over the last 20 years to find a treatment [3] suggests new multifaceted approaches to TBI treatments are required [4, 5].

Hippocampal neurogenesis, a component of cognitive recovery from TBI [6–15], is regulated by key hypothalamic-pituitary-gonadal (HPG) hormones including gonadotropins and sex steroids (reviewed in [16–18]). However, primary hypopituitary hypogonadism associated with TBI can markedly suppress circulating gonadotropin and sex steroid concentrations [19–31]. Hypogonadotropic hypogonadism as a result of acute (≤ 80% of cases) and/or chronic (~40% of cases) TBI could therefore greatly compromise neuroregeneration and recovery from a TBI. In this respect, it is well recognized that the endocrine dyscrasia that results from menopause and during andropause has a negative impact on cognitive function, and that hormone supplementation with physiological human sex steroids can halt or reverse the cognitive decline associated with aging [16, 17]. These data indicate the need to develop strategies to rebalance the HPG hormones following TBI.

Long-chain polyunsaturated fatty acids, like conjugated linoleic acids (CLA), are required for normal neural development and cognitive function [32]. CLA’s are derivatives of linoleic acid (18:2n-6) with conjugated double bonds in the cis or trans configurations [33]. Although many positional and geometric isomers of CLA exist, cis-9,trans-11 CLA (9,11 CLA) and trans-10,cis-12 CLA (10,12 CLA) are the most biologically active forms present in the diet of humans [34] and constitute a small, but significant, component of fats derived from the meat and milk of ruminant animals [35]. While little is known about CLA’s impact on brain function, recently Gama and colleagues [36] demonstrated that as compared to feeding rats a control or a low CLA-enriched butter diet, feeding rats a diet high in CLA increases hippocampal mRNA levels and activity of specific (i.e., iPla_2_g6γ; cPla_2_g4a, sPla_2_g3, sPla_2_g1b, and sPla_2_g12a) phospholipase A_2_ (PLA_2_) encoding-genes in rat brain tissue, which was further correlated with improved memory retrieval performance in the inhibitory avoidance tasks [36]. *In vitro*, Lee and colleagues [37] have shown that CLA treatment of SH-SY5Y cells was protective against H_2_0_2_ and Aβ1–42, inhibited Aβ oligomerization and decreased tau phosphorylation and proapoptotic proteins.

CLA treatment has been shown to stimulate testosterone (T) biosynthesis in the rat Leydig tumor cell line (R2C) by up-regulating CYP17A1 expression [38, 39]. In adult mice, gavage administration of CLA did not increase circulating T concentrations in sedentary mice, but did increase circulating T concentrations above that induced by exercise alone (6 weeks of progressive rotarod training) [39]. Similar to mice [39], in adult human males, CLA intake is linked to increased T biosynthesis following resistant exercise, but not in sedentary individuals [38]. Since consumption of CLA may promote neurogenic sex hormone production, in this study we tested if CLA administration promoted cognitive recovery following a TBI in adult male Sprague Dawley (SD) rats. We find that 1-month treatment of CLA does not reverse craniectomy or CCI injury-induced hypogonadism but does reverse hypoadrenalism, is detrimental to medium- and long-term spatial learning and memory, and limits cognitive recovery following a moderate-severe controlled cortical impact (CCI) injury.

## Materials and Methods

### Subjects

Sprague Dawley (SD) rats (n = 34, 5–6 month old) were acquired from Harlan Laboratories Inc. (Madison, WI) and acclimated to the environment over 2 days. Rats were then weighed and handled for no less than 5 min. each for 5 consecutive days and daily thereafter while being housed, fed and maintained on a 12-hour reverse light/dark cycle. The Institutional Animal Care and Use Committee (Animal Component of Research Protocol) at the William S. Middleton Veterans Administration Hospital approved the procedures used in this study and the research was conducted in an AAALAC-approved facility. Of the 34 rats, 2 rats (1 Sham, 1 CCI) died due to surgical complications and another 3 rats (1 sham, 2 CCI) were excluded for non-compliance during (baseline) behavioral training.

### Surgeries

All surgical procedures were carried out under isoflurane gas anesthesia (5% for induction; 1.5–3% for maintenance). An anesthesia chamber was used for induction and a nose cone was used for maintenance.

#### Controlled cortical impact (CCI) and sham surgeries

Anesthetized rats were mounted in a Kopf stereotaxic device (model 900), where the animal’s head was held in place by non-traumatic ear bars and a bite bar. Anesthesia was maintained by nose cone while the head was shaved and sterilized with 70% ethanol and Betadine^™^ antiseptic solution. Throughout surgery anesthesia levels were monitored closely and were frequently adjusted as need, based on heart rate, respiration rate and oxygen saturation. A homeothermic blanket control unit (Harvard Apparatus, Holliston, MA) was used to monitor body temperature and to prevent hypothermia throughout surgery.

Under aseptic conditions, the cranium and its bony landmarks including bregma (β) and lambda (λ) were exposed by making a midline incision along the scalp into the skin and fascia covering the skull. A 6 mm diameter craniectomy was centered on the midline at 2.5 mm anterior to β. The cortical impact was made at 2.5 mm anterior to β over the midline of the medial frontal cortex with an Impact One^™^ Stereotaxic CCI instrument (Leica), using a 5 mm impactor (bit size), traveling at 2.25 m/s (velocity), extending 3 mm below the cortical surface (impact depth) for 100 ms (dwell time). Sham-injured groups received the same surgical procedures up to and including craniectomy but no CCI injury. After surgery, the rats were placed on a heating pad, monitored closely and upon awakening were tested 30 min. later for Righting Reflex to assess any immediate effects of craniectomy or CCI injury on righting ability, and then returned to their home cages.

### CLA Administration

Rats were assigned to the following groups: Sham + saline group (n = 5), Sham + CLA group (n = 7), CCI + saline group (n = 8), CCI + CLA group (n = 9). CLA groups received intraperitoneal injections of 80% CLA dietary oil (Clarinol^®^ G-80, 25 mg/kg body weight, Stepan Lipid Nutrition USA, Maywood, NJ) and saline groups received intramuscular injections of saline every other day for 28 days (PID’s: 0, 2, 4, 6, 8, 10, 12, 14, 16, 18, 20, 22, 24, 26, and 28). CLA dose was based on 1) current daily recommended intake guidelines (FDA), 2) previous studies in rats examining intracerebroventricular (ICV) administration of CLA on food intake/weight gain [40], dietary CLA’s induction of testosterone synthesis *in vivo* [38] and dietary CLA’s effect on inhibitory avoidance in rats [36], 3) dose used in mice by Sato and colleagues [41], and 4) extrapolation from mouse work performed by our co-author Dr. Ian Bird. Clarinol^®^ G-80, contains triglycerides—predominantly c-9,t-11 (conjugated diene (CD)18:3, CD20:3) and t-10,c-12 (CD20:4) CLA, with traces of oleic, palmitic and stearic acid (or safflower oil fatty acids). Animal IDs were coded to keep experimenters blind to group identity throughout behavioral testing and histological analysis.

### Transcardiac Perfusion

Surgeries were performed on post-injury day (PID) 29. At the time of sacrifice, rats were placed under 5% isoflurane for >10 min. and then the heart was exposed via thoracotomy, and an incision was then made in the left ventricle in which a blunted butter fly needle (attached to a Perfusion-One system) was inserted and clamped in place. After making an incision in the right atrium, the Perfusion-One system (set at 100–150 ml/min) was turned on to transcardially perfuse with cold saline for 10–15 min. After infusing saline, the rats were sacrificed by decapitation and the brain removed and imaged for the front, side and top views.

### Cognitive, Behavioral and Motor Testing

#### Righting reflex

The righting reflex was conducted on each rat 30 min. after awaking from anesthesia following Sham and CCI. Righting reflex was deemed compromised if a rat did not right itself within 15 s of being placed on its back [42, 43].

#### Anxiety-like behavior in the elevated plus maze

In the elevated plus maze (EPM), anxiety is computed by determining the amount of time spent in the open vs. the closed arms [44]. This is because in novel situations, rats tend to exhibit thigmotactic behavior (i.e. huddling next to walls or enclosed spaces which provide mechanical stimulation). Because laboratory rats are naturally exploratory, reduced thigmotaxis (inferred from time spent exploring the open arm) is taken as an indication of lowered stress or anxiety [45]. Thus the total number of open vs. closed arm entries was used as the operational dependent measure. If the animal fell off the open arm it was returned to the start position in the center square and the fall recorded. Individual trial data for each rat was used to determine group averages. Falls were very infrequent (1 out of 30 rats).

Testing was conducted under red light in a quiet environment. Baseline EPM data was obtained on PID -7 and rats were tested again on PID’s 2, 10, 15, and 21. Each trial lasted 5 min., and the total number of open arm entries (or crosses over the center square with both front and hind paws) was reported as percent of visits to the open and closed arms.

#### Rotarod testing of vestibular balance and coordination

An accelerating Rotamex^™^ rotarod (Columbus Instruments, Columbus, OH) was used under red light to assess balance and motor coordination [46]. Rats were given initial habituation training and then baseline testing prior to surgery. For habituation training, rats were placed onto a stationary rod for at least 3 min., then slowly habituated to the rotating rod from a starting speed of 1 rpm, which was accelerated to 30 rpm over 5 min. A baseline score was obtained on PID -3 and rats were again tested on PID’s 2, 6, 9, 13 and 23. Rats were scored on latency to fall off the rod (maximum score was 300 s), and each testing day consisted of 3 trials separated by a 10-min. break. Scores from the three trials for each day were averaged.

#### Spatial navigation performance and memory in the MWM

MWM tests were conducted under dim white light in a white plastic pool measuring 135 cm in diameter [47]. The rat’s position in the maze, swim distance, and latency to reach the platform was recorded with an overhead camera and computer-assisted tracking system (CleverSys, Reston, VA). Beginning on PID 6, all the rats underwent 5 consecutive days of ‘‘Acquisition training’ in the MWM. Each animal received two trials per day, separated by a 5min. interval. A trial consisted of placing the subject in the pool facing the wall (NE starting point). The rats were allowed to swim until they reached the platform or until 90 s had elapsed. When rats were unable to locate the MWM platform within the allotted time, the experimenter led the rat to the platform. Rats were allowed to remain on the platform for 20 s and then removed from the pool. After 5 min., subjects were again released into the tank, but this time from a different starting position (SE starting point) from the previous trial and allowed to swim to the platform. Animals were placed in holding cages in front of an air heater between trials and before being returned to their home cages. Forty-eight hours after the last acquisition trial, the MWM platform was removed from the pool and each rat was given a Probe test, in which they were allowed 60 s to swim around the MWM tank and the amount of time spent in each quadrant (N, S, E and W) were calculated as the dependent measure. After the first probe test, the MWM platform was returned to the pool (in a different quadrant) and the ‘Re-acquisition Phase’ occurred on PID’s 21–23, in which, rats had to learn to find the hidden MWM platform in its new location. A second Probe test was conducted (on PID 25), 48 h after the last re-acquisition trial. Time spent in each quadrant was recorded as the dependent measure. We have previously published data [47] demonstrating that this particular MWM design (i.e., 5 days of 2-run acquisition trials and 3 days of 2-run reversal learning) was sensitive and sufficient enough to detect CCI-induced functional deficits in juvenile male Sprague Dawley rats and the effectiveness of progesterone treatment (i.e., 9 injections over 7 days) was also detectable when using this MWM testing paradigm.

### Histology

Whole brain images were taken with a Nikon digital camera and the percent of damaged tissue was identified using the Image J^™^ System (Media Cybernetics, Silver Spring, MD). The percentage of injured tissue for each rat was calculated by tracing the perimeter of the injured area and the determining its surface area, compared to the diameter of the whole cerebral cortex.

### Blood Collection and Hormone Analyses

Rats were anesthetized and their tails placed in a 200 mL beaker filled with warm water (≤44°C) for 5 min. Each tail was clean with 70% alcohol, the tip snipped with scissors, and 2 mL of whole blood was collected directly into EDTA tubes (PID’s -10, 1, 11, 19 and 29). At the terminal bleed (day 29), blood also was collected via heart puncture. Collected blood was immediately centrifuged at 4,000*g* for 10–20 min. and the plasma aliquoted into Eppendorf tubes for storage at -80°C. Plasma samples were analyzed at the Assay Services Laboratory in the Wisconsin National Primate Research Center of the UW-Madison Institute for Clinical and Translational Research for progesterone (P_4_), T, 11-deoxycorticosterone (11-DOC) and corticosterone adapted from a method previously described [48, 49]. Briefly, to plasma samples (400 μl) internal standard (200 pg d9-progesterone and d5-testosterone and 1 ng d4-cortisol) was added and the samples were extracted with methyl tert butyl ether. The organic phase was transferred to a clean vial and evaporated to dryness and then a second dichloromethane extraction was performed. The organic phase was transferred into a clean test tube and evaporated to dryness and reconstituted in mobile phase. Samples were analyzed on a QTRAP 5500 quadruple linear ion trap mass spectrometer (AB Sciex) equipped with an atmospheric pressure chemical ionization source. The system includes two Shimadzu LC20ADXR pumps and a Shimadzu SIL20ACXR autosampler. A sample of 30 μl was injected onto a Phenomenex Kinetex 2.6u C18 100A, 100 × 2.1 mm column for separation using a mobile phase: water with 1% formic acid (Solution A) and acetonitrile with 1% formic acid (Solution B), at a flow rate of 200 μl/min. Quantitative results were recorded as multiple reaction monitoring (MRM) area counts after determination for the response factor for each compound and internal standard. Each steroid had a MRM used for quantitation and 1 or 2 additional MRMs as qualifiers. The linearity was *r* > 0.9990 and the curve fit was linear with 1/*x* weighting. None of the compounds of interest were detected in blank or double blank samples. Inter-assay coefficient of variation was determined from a pool of human serum and ranged from 6.09–19.47%.

### Statistical Analysis

A mixed factorial analysis of variance (ANOVA) for repeated measures was performed on the weight, behavioral, hormonal and gross lesion data (GraphPad Prism, v.7; GraphPad Software, Inc., La Jolla, CA). Mean comparisons were used for *post-hoc* analyses of repeated ANOVAs. Independent paired *t*-tests were also used to compare the differences between baseline (pre-injury) and post-injury data when data were normally distributed. Statistical significance was established at *p* ≤ 0.05.

## Results

### CLA Does Not Reverse Craniectomy- or Craniectomy + CCI Injury-Induced Hypogonadism but Does Reverse Hypoadrenalism

**Fig 1:**
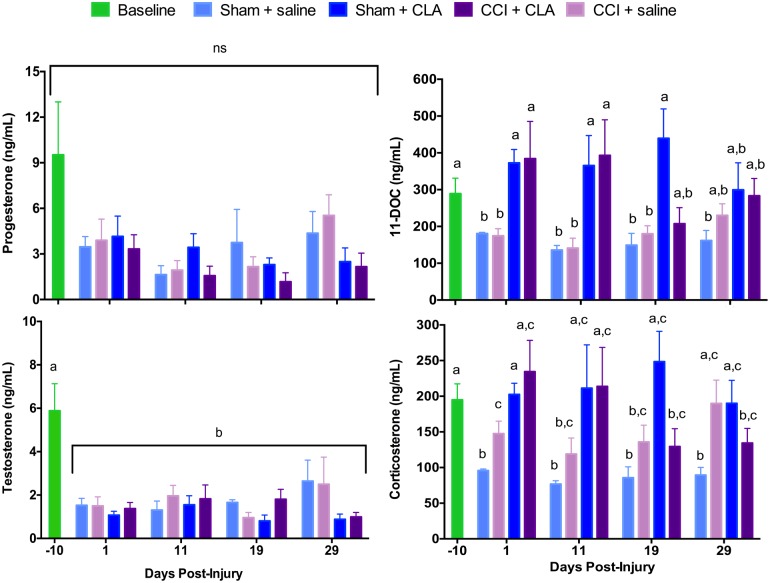
CLA does not restore CCI-induced decreases in circulating sex hormone concentrations. Plasma concentrations (mean ± SEM) of P_4_, T, 11-DOC and corticosterone (ng/mL) at PID’s -10, 1, 11, 19 and 29 for Sham + saline (n = 5), Sham + CLA (n = 5), CCI + saline (n = 8) and CCI + CLA (n = 5) groups. Data were analyzed using 2-way repeated measures ANOVA; post-hoc analyses were performed using the Tukey multiple comparison test (p < 0.05, letters indicate differences between post-injury days and treatments for T, 11-DOC and corticosterone; for P_4_, results did not reach significance for main effect of post-injury day or treatment, p = 0.068).

Sham surgery (craniectomy alone; Sham + saline group) induced a significant decline from baseline in the circulating concentrations of P_4_ (64.2%), T (74.6%), 11-DOC (37.5%) and corticosterone (50.8%) by PID 1 (Fig 1). Similar declines in circulating concentrations of P_4_ (58.9%), T (74.6%), 11-DOC (39.4%) and to a lesser extent corticosterone (24.6%) were observed for CCI injured animals (CCI + saline group) on PID 1, indicating that surgery alone was sufficient to induce hypogonadotropic hypogonadism and hypoadrenalism. Circulating concentrations of P_4_ and T in Sham surgery and CCI injured animals remained at this lower concentration through PID 29. Circulating concentrations of 11-DOC for both Sham + saline and CCI + saline groups, and corticosterone for the Sham + saline group, also remained low through till PID 29, while corticosterone in the CCI + saline group was decreased, but only significantly at PID 11.

CLA treatment did not increase circulating concentrations of either P_4_ or T in Sham or CCI injured animals through PID 29 (Fig 1). CLA increased circulating 11-DOC concentrations from PID 1 through PID 29 in both Sham and CCI injured animals, although this effect was diminished by PID 29. CLA increased circulating corticosterone concentrations from PID 1 through PID 29 in Sham animals, but this effect was only observed on PID1 and PID 11 for CCI injured animals.

### CCI and CLA Have Detrimental Effects on Cognitive Performance

Righting reflex performance did not differ between animals in any group after surgery. CCI injury or CLA treatment did not impact anxiety-related activity levels as assessed via the elevated plus maze on PID’s 2, 10, 15, and 21 (data not shown).

To test spatial learning and memory, rats were trained in MWM performance and latency to reach a hidden platform was recorded over 5 days (Acquisition Phase), after which, spatial memory was tested by moving the platform to a novel location (Re-acquisition Phase), or out of the MWM tank (Probe Tests). Analysis of swim speeds (distance travelled/latency to platform), during Run 1 on the 5 acquisition and 3 re-acquisition trial days, indicated that there were no significant differences between Sham + saline and Sham + CLA or between CCI + saline and CCI + CLA (as determined via a 2-way repeated measures ANOVA; Main Effect of Experimental Group: F(4,38) = 0.791, p = 0.5385 (where α = 0.05); Main Effect of Test Day, F(7,266) = 0.68, p = 0.69; Interaction, F(28,266) = 0.59, p = 0.95)). These data imply that latency to reach the platform would be minimally affected, as a dependent measure. We therefore present MWM results as latency to reach platform, as opposed to distance travelled.

**Fig 2:**
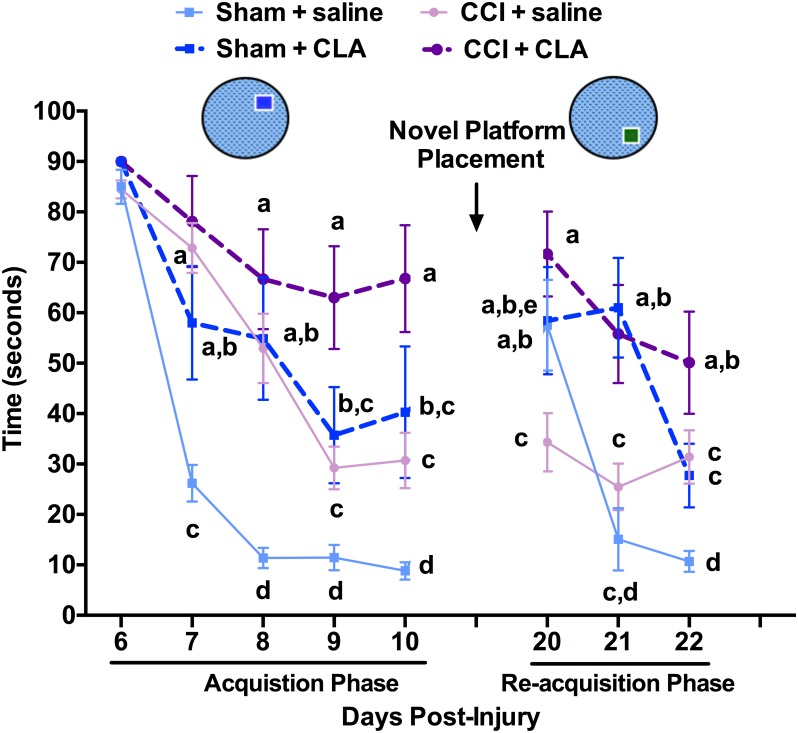
Chronic CLA administration compromises medium-long term learning and memory in both uninjured and CCI-injured rats as assessed by latency to locate the Morris water maze (MWM) platform. First Run: Latency in seconds to reach the hidden platform at PID’s 6–10 (Acquisition Phase; platform in SE quadrant) and at PID 20–22 (Re-acquisition Phase; platform changed to NE quadrant, ‘Novel Platform Placement’). MWM Run 1 latency data (mean ± SEM) on PID’s 6–10 and 21–22 were analyzed from 29 rats as follows: Sham + saline (n = 5), Sham + CLA (n = 7), CCI + saline (n = 8) and CCI + CLA (n = 9). Data were analyzed using 2-way repeated measures ANOVA; post-hoc analyses were performed using the Tukey multiple comparison test (p < 0.05; a, b, c and d = differences between time and group, e = differences within groups between the acquisition (PID 10) and re-acquisition phases (PID 20)).

We found no difference between groups in latency to reach the platform on day 1 (PID 6) of the acquisition Phase (p > 0.05, Fig 2). By day 5 (PID 10) of the acquisition Phase, animals in the Sham + saline group were the fastest to find the hidden MWM platform (8.8 ± 1.7 s). Animals in the CCI + saline group were significantly slower (30.7 ± 5.5 s at PID 10). Unexpectedly, animals in the Sham + CLA group were just as slow (40.3 ± 13.0 s at PID 10) as the CCI + saline group, while CCI animals receiving CLA were the slowest (66.8 ± 10.2 s at PID 10) and showed no improvement in finding the hidden platform over the 5-day acquisition phase.

Ten days after the acquisition phase (PID 20) the platform was moved from the NE to the SE quadrant (novel platform placement; NPP) and rats were tested for 3 days (re-acquisition phase). Time to find the hidden platform in its new location was significantly increased 6.5-fold in Sham + saline animals (from 8.8 ± 1.7 s (PID 10) to 57.5 ± 9.0 s (PID 20), *p < 0*.*05*) but was not increased in Sham + CLA animals (40.3 ± 13.0 s to 58.4 ± 10.6 s, *p > 0*.*05*), indicating that CLA treatment did not allow the retention of long-term memory of the previous platform location. Animals in the CCI group treated with saline (30.7 ± 5.5 s to 34.3 ± 5.8 s, *p > 0*.*05*) also did not retain long-term memory of the previous platform location (NE quadrant; Fig 2), while animals in the CCI + CLA group (66.8 ± 10.6 s to 71.7 ± 8.4 s, *p > 0*.*05*) remained oblivious to the platform location. After one NPP trial, animals in the Sham + saline group quickly learned the new location of the hidden platform (i.e. SE quadrant) such that latency to find the platform improved (15.1 ± 6.2 s, PID 21) to that at the end of the (NE) acquisition phase (8.8 ± 1.7 s, PID 10; Fig 2). However, CLA treatment of Sham animals prevented learning of the new SE location with the latency to find the platform (61.0 ± 9.9 s; PID 20) being similar to that at the end of the NE acquisition phase (40.3 ± 13.0 s; PID 10). Similar to the Sham + CLA group, there was no improvement in latency to find the platform in the CCI groups during the SE re-acquisition phase. In the CCI group, CLA treatment exacerbated the effect of the CCI injury during the NE acquisition phase, resulting in a two-fold increase in the amount of time taken to find the hidden platform (66.8 ± 10.6 s; PID 10), when compared to that of the saline treated group (30.7 ± 5.5 s; PID 10). Together, the Sham + CLA and the CCI + CLA results indicate that immediate and chronic CLA is detrimental to medium-long-term spatial learning and memory in rats whether uninjured (craniectomy only) or following a moderate-severe CCI (craniectomy + CCI injury).

**Fig 3:**
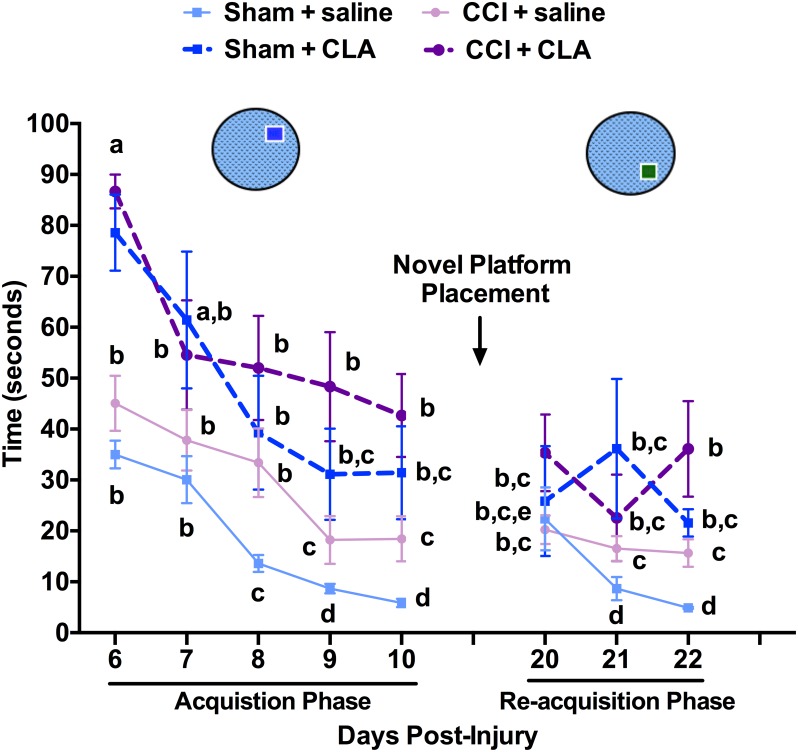
Chronic CLA administration compromises short-medium term learning and memory in both uninjured and CCI-injured rats as assessed by latency to locate the Morris water maze (MWM) following novel platform placement. Second Run: Five minutes after Run 1 (see Fig 4), latency in seconds to reach the hidden platform at PID 6–10 (Acquisition Phase; platform in SE quadrant) and at PID’s 20–22 (Re-acquisition Phase; platform changed to NE quadrant, ‘Novel Platform Placement’) was tested. MWM Run 2 latency data (mean ± SEM) on PID’s 6–10 and 21–22 were analyzed from 29 rats as follows: Sham + saline (n = 5), Sham + CLA (n = 7), CCI + saline (n = 8) and CCI + CLA (n = 9). Data were analyzed using 2-way repeated measures ANOVA; post-hoc analyses were performed using the Tukey multiple comparison test (p < 0.05; a, b, c and d = differences between time and group, e = differences within groups between the acquisition (PID 10) and re-acquisition phases (PID 20)).

In the acquisition trial conducted 5 min. after the first run, all groups showed similar trends in improvement over the next 5 days (Fig 3). However, while Sham + saline and CCI + saline animals remembered the NE platform location from the first acquisition trial as indicated by the reduced time to find the platform on PID 6 (85.0 ± 3.4 s vs. 35.0 ± 2.7 s and 84.5 ± 1.8 s vs. 45.1 ± 5.4 s; PID 6 run 1 vs. run 2, respectively), the Sham + CLA and CCI + CLA animals did not remember the same NE platform location from the first acquisition trial on PID 6 (90.0 ± 0.0 s vs. 78.6 ± 7.5 s and 90.0 ± 0.0 s vs. 86.7 ± 3.3 s; PID 6 run 1 vs. run 2, respectively). The short-medium term memory of CLA treated animals was clearly compromised compared with that of untreated animals. As for the re-acquisition phase, Sham + saline and CCI + saline animals remembered the SE platform location from the first acquisition trial (57.5 ± 9.0 s vs. 22.4 ± 6.2 s and 34.3 ± 5.8 s vs. 20.2 ± 2.8 s; PID 20 run 1 vs. run 2, respectively). Interestingly, the SE platform location from the first acquisition trial was remembered by the Sham + CLA animals (58.4 ± 10.7 s vs. 25.9 ± 10.8 s; PID 20 run 1 vs. run 2, respectively) and the CCI +CLA animals (71.7 ± 8.4 s vs. 35.3 ± 7.5 s; PID 20 run 1 vs. run 2, respectively). These results indicate that CLA did not impact short-medium term memory during the reacquisition phase in Sham animals, and did not improve short-medium term memory in CCI-animals.

**Fig 4:**
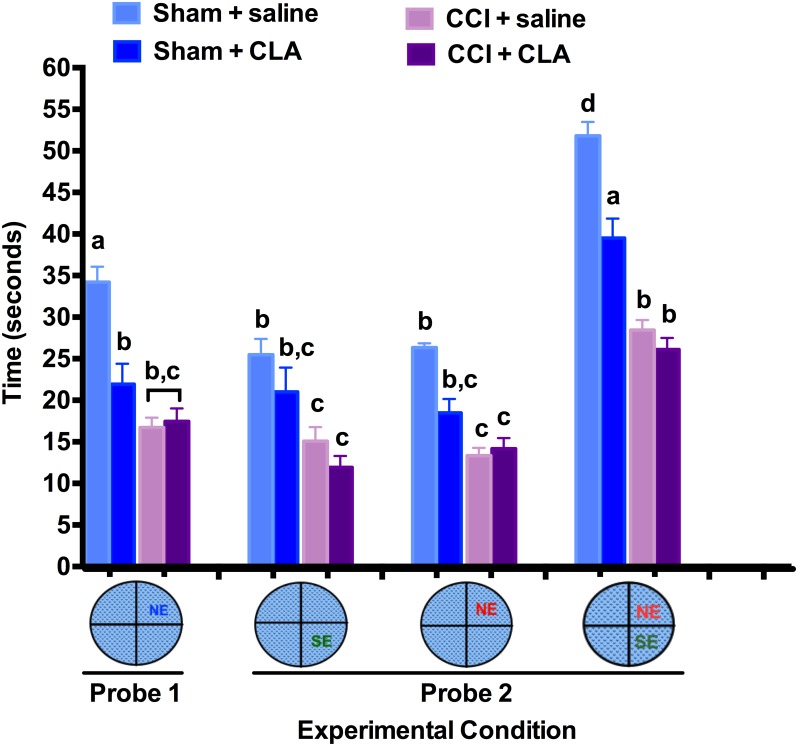
Chronic CLA administration is detrimental to medium-long term spatial memory. Probe Test 1: At the end of each acquisition phase, the platform was removed (from the NE quadrant) and 48 h later the animals were tested in the MWM for NE quadrant preference over 60 s to determine if they remembered the location of the hidden platform. Probe Test 2: Likewise, after completing the re-acquisition phase the platform was removed from the SE quadrant and 48 h later the animals were tested for SE (new location), NE (old location) and SE + NE quadrant preferences (both new and old locations). MWM duration in target quadrant(s) data (mean ± SEM) during Probe Test 1 and 2 were analyzed from 29 rats as follows: Sham + saline (n = 5), Sham + CLA (n = 7), CCI + saline (n = 8) and CCI + CLA (n = 9). Data were analyzed by ANOVA; post-hoc analyses were performed using the Tukey multiple comparison test (p < 0.05; a, b, c and d = differences between time and group, e = differences within groups between the acquisition (PID 10) and re-acquisition phases (PID 20)).

At the end of each acquisition phase, the platform was removed and 48 h later the animals were tested in the MWM for quadrant preferences over 60 s to determine if they remembered the location of the hidden platform (Probe test 1; NE quadrant). CLA treatment of Sham animals significantly decreased the amount of time spent in the NE quadrant (21.0 ± 2.9 s) compared to saline treated Sham animals (34.2 ± 1.8 s). Compared with the Sham group (34.2 ± 1.8 s), CCI significantly decreased the amount of time spent in the NE quadrant (16.7 ± 1.2 s) to that of chance finding (15 s), an effect that was **not** reversed by CLA administration (17.5 ± 1.6 s; Fig 4). This result indicates that immediate and chronic CLA does not improve medium-term memory following a moderate-severe CCI.

Similar to Probe test 1, 48 h after completing the re-acquisition phase the platform was removed and the animals were tested for quadrant preferences (Probe test 2). CCI reduced the preference for the new SE quadrant as well as the former NE quadrant. CLA treatment did not significantly impact the time spent in the newer SE quadrant (platform placement 2 days prior) or time spent in the older NE quadrant (platform placement 12 days prior). CLA did not alter time spent in the SE quadrant by Sham animals (21.0 ± 2.9 s) compared to the Sham saline group (25.5 ± 1.9 s, *ns*).

Analysis of the accumulated time spent in both quadrants associated with the hidden platform indicated that CLA treatment significantly reduced the time (39.5 ± 2.4 s) spent by Sham animals in the associated quadrants compared with saline treated Sham animals (51.8 ± 1.7, *p < 0*.*05*). CCI animals treated with saline spent less time (26.1 ± 1.4 s) in the quadrants associated with the hidden platform compared with the Sham animals, and CLA treatment did not change the time spent by CCI animals in the platform associated quadrants (28.4 ± 1.0 s, *p > 0*.*05*), with both groups performing at the level of chance (~30 s). The probe tests indicate that immediate and chronic exposure to CLA is detrimental to medium- and long-term spatial memory.

### CLA Does Not Improve CCI-induced Declines in Vestibulomotor Performance

**Fig 5:**
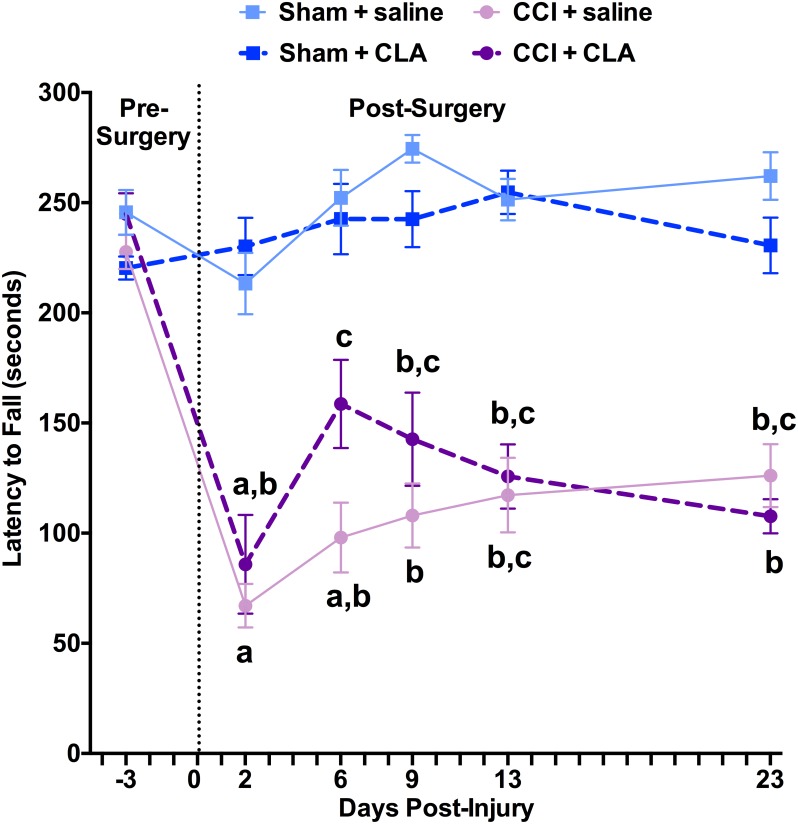
CLA does not improve vestibulomotor function following CCI. Rats were placed on a rotating rod accelerating at a constant rate (1 rotation per second per second) and allowed to run for up to 300 s at PID’s -3, 2, 6, 9, 13 and 23. Time elapsed (seconds) before fall was recorded. Rotarod latency data (mean ± SEM) on PID’s 6–10 and 21–22 were analyzed from 29 rats as follows: Sham + saline (n = 5), Sham + CLA (n = 7), CCI + saline (n = 8) and CCI + CLA (n = 9). Data were analyzed using 2-way repeated measures ANOVA; post-hoc analyses were performed using the Tukey multiple comparison test; letters indicate differences between groups and times compared to baseline and sham groups, *p* < 0.05.

To test vestibulomotor performance, rats were trained on a Rotarod. Latency to fall from the Rotarod was not significantly different between groups at PID -3 (Sham + CLA: range from 220 ± 5 to 246 ± 10 s; Fig 5). Latency to fall from the Rotarod was significantly decreased in CCI rats treated with saline (67 ± 10 s; *p < 0*.*05*) or CLA (86 ± 22 s; *p < 0*.*05*) on PID 2 by ~150 s compared to sham rats treated with saline (213 ± 14 s; *ns*) or CLA (230 ± 13 s; *ns*), and remained significantly decreased throughout the remainder of the experiment (i.e., PID’s 6, 9, 13 and 23, p < 0.05). CLA treatment did transiently improve rotarod performance in the CCI group compared to CCI saline group on PID 6 (159 ± 20 s vs. 98 ± 16 s, respectively; p < 0.05). However, this benefit was lost over the following 17 days ((108 ± 7 s vs. 126 ± 14 s), respectively; ns). These results indicate that CLA does not improve vestibular balance and motor coordination in rats following CCI.

### CLA Does Not Reverse CCI-induced lesion Size

**Fig 6:**
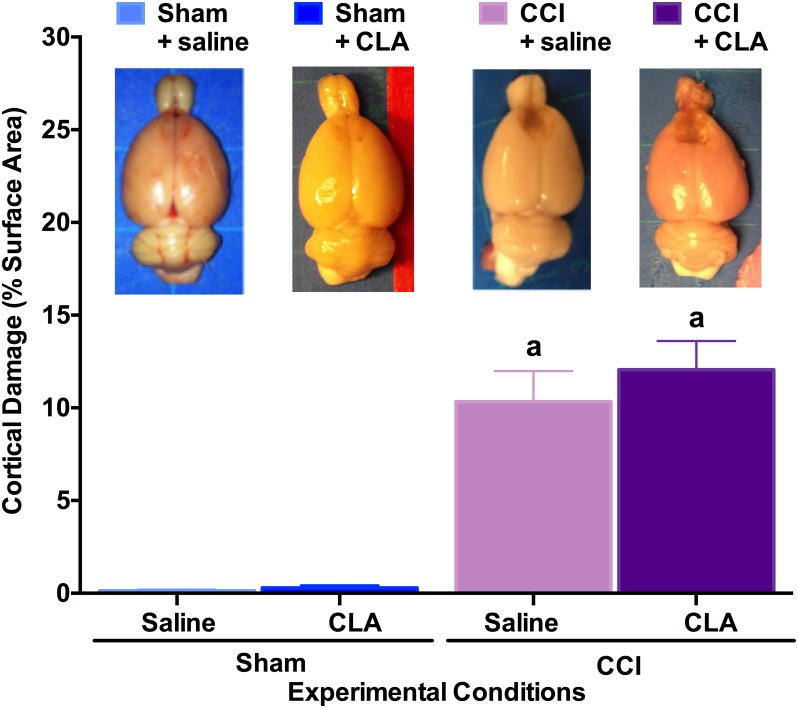
CLA does not reduce gross lesion size following a CCI. Top: Representative figures for each group, illustrating lesion size 1-month post-CCI. Bottom: Lesion size depicted as a percentage of surface area damaged (y-axis) for rats in each surgery/treatment group (x-axis). Gross lesion size data (mean ± SEM) at PID 29 were analyzed from 29 rats as follows: Sham + saline (n = 5), Sham + CLA (n = 7), CCI + saline (n = 8) and CCI + CLA (n = 9). Data were analyzed using ANOVA; post-hoc analyses were performed using the Tukey multiple comparison test (p < 0.01; letter indicates differences between groups).

Post-mortem analysis of brains indicated that rats with CCI had significant tissue damage as a percent of total cortical area compared to Sham groups (10.3 ± 1.7%; p = 0.05; p < 0.01), while immediate and chronic CLA treatment did not reduce gross lesion size in adult male SD rats with CCI injury (12.1 ± 1.6%; p > 0.05; Fig 6).

### CLA Does Not Reverse CCI-induced Reductions in Body Weight

**Fig 7:**
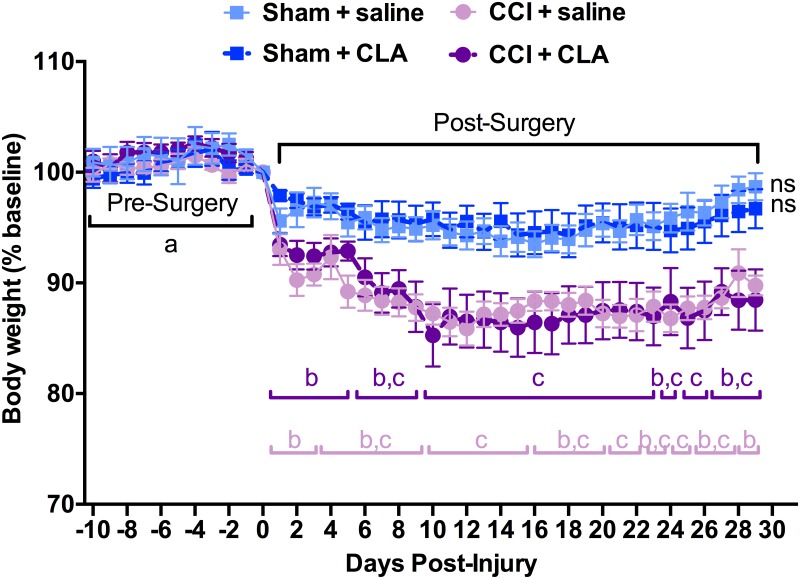
CCI, but not CLA, reduces body weight. Body weight (as a % of baseline weight) each day from PID -10 to PID 29 for Sham + saline (n = 5), Sham + CLA (n = 7), CCI + saline (n = 8) and CCI + CLA (n = 9) groups. Data were analyzed using 2-way repeated measures ANOVA; post-hoc analyses were performed using the Tukey multiple comparison test (p < 0.05; letters indicate differences between pre- and post-surgery body weights between groups across the experiment).

Body weight pre-surgery (i.e., from PID -10 to PID -1) varied by less than 2.6% and did not differ from baseline (PID 0) for any of the 4 groups (Fig 7). The weights of Sham-operated saline-treated rats and of Sham-operated CLA treated rats did not change from baseline (PID 0, 498 ± 19 g and 635 ± 13 g, respectively) over the 29 days. CCI injury reduced mean body weight in saline-treated rats by 6.7% within 2 d post-surgery (i.e., from 502 ± 26 g on PID 0 to 461 ± 25 g on PID 2, *p < 0*.*05)*, by 13.7% on PID 12 (i.e., 433 ± 24 g) and 10.8% at PID 29 (i.e., 463 ± 23 g). CLA treatment did not alter body weight in CCI injured animals except for PID 5 where there was a ~5% increase (c = p <0.05) in mean body weight between this group and the CCI + saline treated rats.

## Discussion

We demonstrate for the first time that intraperitoneal injection of Clarinol^®^ G-80 is detrimental to medium- and long-term learning and memory in young adult male rats (Figs 2, 3 and 4). In addition to acting like an amnesic agent in uninjured rats, we also show for the first time that Clarinol^®^ treatment exacerbates cognitive recovery in rats following an acute focal CCI injury (Figs 2, 3 and 4). These results suggest that a diet high in CLA should be avoided for optimal cognitive performance or recovery from a TBI. CLA did not affect vestibular performance in uninjured adult male rats and while CLA did improve vestibular performance briefly following a CCI injury (PID 6) this effect disappeared by PID 9 (Fig 5). CLA was ineffective at reducing TBI-induced neuropathology following an acute focal CCI injury (Fig 6).

Only a few papers have examined the impact of CLA on cognitive function. A recent short-term study in humans demonstrated no change in cognitive performance in elderly men and women supplemented with CLA for 8 weeks with the exception of improving auditory verbal learning in men [50]. In contrast, Gama and colleagues [36] demonstrated that a diet high in CLA’s enhanced performance in an inhibitory avoidance memory task in Wistar rats. Animal studies have shown that 85–100% of the ingested CLA isomers are absorbed from the gut and distributed mainly to adipose tissue, brain, heart, liver, lung, kidney, spleen, adrenals, and in serum [51]. Fa and colleagues [52] found that a bolus oral gavage administration of CLA (2g), in female Sprague Dawley rats, resulted in peak levels in plasma (favoring the 10,12 isomer) and liver accumulation of the two CLA’s after 12 h (350–450 and 50 nM/mg of lipids), while brain and adipose (favoring the 9,11 isomer) tissue levels peak at 24 h (2.0 and 5–10 nM/mg of lipids). The transfer of CLA’s across the blood-brain barrier [52] has been shown to 1) reduce cerebral prostaglandin E2 [53], 2) prevent angiogenesis [54], 3) protect against glutamate excitotoxicity (cis-9, trans-11 isomer) [55] and 4) increase hippocampal mRNA levels and enzymatic activity for specific phospholipase A2 (PLA2) isoforms [36]. While CLA’s (positive or negative) effects on learning (encoding) and/or memory (retrieval) via the above mechanisms is currently unknown, it is clear that CLA is linked to calcium independent PLA2 activity, which is in turn, is required for short- and long-term memory formation [56], negatively correlated with the density of neurofibrillary tangles [57], and plays distinct roles in apoptosis and neurogenesis [58]. It would appear given that medium term (5-min) memory is compromised (compare PID 6 time points for CLA treated and untreated animals; Figs 2 and 3), that this effect on medium-term memory is not mediated via new protein synthesis but by a change in enzymatic activity (i.e. PLA2?).

Another mechanism may involve cell cycle dynamics. The maintenance of longer-term memories has been associated with the presence of sex steroids [59] and neurogenesis in the adult brain [60]. CLA’s have significant impacts on cell proliferation and differentiation, as evidenced by their effects on mammary ductal elongation and terminal end-bud formation (mediated via IGF-1; [61]), and its suppression of chemical carcinogen-induced mammary tumor burden in rats [62, 63]. In this respect, increasing neurogenesis after the formation of a memory has been shown to be sufficient to induce forgetting in adult mice [60]. However, in contrast, during infancy, when hippocampal neurogenesis levels are high and freshly generated memories tend to be rapidly forgotten (infantile amnesia), decreasing neurogenesis after memory formation has been shown to mitigate forgetting. In addition to neurogenesis-related impacts on memory formation, the dietary 10,12 CLA isomer can elicit inflammatory responses that includes the recruitment of immune cells such as macrophages and mast cells, and an inflammatory state at both the systemic and tissue levels that manifests as decreased serum adiponectin and elevated IL-6 and tumor necrosis factor-α mRNA in adipose tissue (reviewed in [61]). Inflammation is associated with memory loss in Alzheimer’s disease. However, our study used both 9,11 and 10,12 isomers that generally as a mixture tend to have less adverse outcomes than 10,12 alone. CLA’s impact on neurogenesis and neuroinflammation warrant further investigation.

Clarinol^®^ (<3.4 g/day) is approved safe for long-term use by the US Food and Drug Administration (FDA, 2004) and the European Food Safety Authority (EFSA, 2012). In humans basal circulating 9:11 CLA is ~5 μM while 10,12 is undetected [41, 64]. Eating 2g/day of commercially available CLA, in which the 9,11 and 10,12 isomers are predominant and present in approximately equal amounts [65], is enough to achieve circulating concentrations of 36 μM and 20 μM of 9,11 and 10,12 CLA, respectively, and is not considered harmful [41]. In our study we injected 25 mg/kg CLA intraperitoneally every other day (~1.625 g/day, assuming 65 kg human). Although this level is half that of the FDA approved dose, it significantly suppressed cognitive performance. The two most abundant CLA isomers (*cis*-9,*trans*-11 and *trans*-10,*cis*-12) have distinct kinetics and metabolism (i.e., conversion to conjugated dienes (CD)18:3 or CD16:2 metabolites), and exert distinct biological effects [65–72]. Further studies are required to determine whether the 9,11 and/or 10,12 CLA isoforms are detrimental to cognitive performance.

### CLA Impact on Body Weight

Mixtures of CLA have become widely available as a weight loss supplement [73], in which the 9,11 and 10,12 isomers are predominant and present in approximately equal amounts [65]. While CLA is increasingly being advertised as a weight loss product [74], and has been shown to modify body mass [75], strong support for the weight reducing effects of CLA in humans [76] and mice [77] is far from unanimous. Since higher doses of CLA (> 10g/day) have been associated with weight loss in humans, our dose may have been insufficient to promote weight loss. Furthermore, the route of administration may impact the magnitude and direction of CLA’s effect. For example, rat pups given a CLA-supplemented diet after weaning had significantly greater body weight gain and improved feed efficiency relative to control animals (P < 0.05) [78], while intracerebroventricular administration of CLA inhibited food intake by decreasing gene expression of neuropeptides Y (NPY) and agouti-related protein (AgRP) [79]. It is important to note that during the times of daily MWM testing (i.e., PID 6 to PID 10), which is presumably a stress-inducing behavioral paradigm, both CCI, but not sham, groups appear to undergo a secondary (≥3.0%) dip in weight loss. The importance and extent of this TBI-induced weight loss on the animal life course requires further investigation.

### CCI Impact on Plasma Steroid Concentrations

The plasma concentrations of P_4_, T, 11-DOC and corticosterone were significantly reduced in both surgery and CCI injured animals (Fig 1), indicating that surgery alone was sufficient to induce hypogonadism and hypoadrenalism. The decline in P_4_ following a TBI was similar to that reported by Wright and colleagues [80]. These results are similar to those reported in humans, where early studies demonstrated that TBI could alter hypothalamic morphology and induce hypogonadism and hypothryoidism [81, 82]. Subsequent studies demonstrated that the post-traumatic hypopituitarism, initially unresponsive to GnRH in severe TBI, could eventually be reversed with GnRH treatment and indicated a hypothalamic cause for hypogonadism [83]. This was confirmed by Woolf and colleagues [84], where the initial decline in sex hormones (T, E_2_, LH and FSH) within 24–48 h post-TBI in men and women (hypogonadotropic hypogonadism) was reversed over time and with GnRH treatment [84]. This group subsequently showed that severe TBI leads to transient hypogonadotropic hypogonadism, which affects T and its precursors (17-hydroxyprogesterone, and DHEA sulphate). Similar to TBI, a transient hypogonadotropic hypogonadism has been observed post-surgery where circulating LH, FSH and E_2_ concentrations are decreased [85]. Lee and colleagues [86] examining severe TBI demonstrated no relationship between the severity of brain injury and the levels of T, FSH or LH suggesting that the hypogonadism may reflect more the stress-induced (cortisol?) suppression of HPG axis function as it does the structural and neurochemical disruption of the axis [86]. In these severe TBI cases, it is likely that any structural disruption to the HP early gives way to its suppression by stress hormones later that eventually resolves allowing for the return of normal circulating sex hormones concentrations.

More recently it has been recognized that the degree and type of neuroendocrine dysfunction, and recovery, following a TBI depends on numerous factors including the severity, frequency and location of the TBI, and the age and sex of the individual [22, 87–97]. Lower total and free T-levels at admission are associated with lower total functional independence measure scores at admission and discharge [29, 30]. More recently, Wagner et al., [31] measuring T and E_2_/T ratios found that acute hypogonadotropic hypogonadism affected 100% of the TBI population, while 37% subsequently developed persistent hypogonadotropic hypogonadism. Those with persistent hypogonadotropic hypogonadism are associated with worse functional and cognitive outcomes at 6- and 12-months post-injury [28, 31, 98]. Circulating T at 12- to 16-weeks post-injury was demonstrated to predict poor outcome 1 year after severe TBI [28].

In our study, T, P_4_ and 11-DOC were suppressed for 29 d following both TBI and surgery, suggesting that the surgery and CCI-injury used in our study produces a longer-lasting suppression of hypothalamic function than the predominantly closed-head injuries discussed above in humans. Corticosterone concentration post-surgery also did not rebound over 29 d, but surprisingly did rebound in post-TBI animals by PID 29, suggesting that perhaps the hypothalamic-pituitary-adrenal axis had recovered following the CCI injury but not following Sham surgery.

### CLA Impact on Plasma Steroid Concentrations

CLA did not reverse hypogonadism induced by surgery or CCI injury; the circulating concentration of the neurogenic sex steroids T (and P_4_) remained unchanged (Fig 1). However, we do report for the first time that CLA treatment did mostly reverse the surgery and CCI injury induced hypoadrenalism; CLA increased the glucocorticoid precursor 11-DOC and the mineralocorticoid precursor corticosterone mostly back to pre-surgery concentrations in both Sham and CCI-injured animals (Fig 1). These differential effects of CLA on circulating sex steroid and corticosteroid concentrations may be due to the attenuating effect of CLA on FSH signaling, aromatase expression and E_2_ production as observed in granulosa cells [99]. This would result in decreased sex steroid production but increased concentrations of steroid precursors available for use in corticosteroid pathways. Whether the CLA-induced elevation in corticosteroids (or alteration in the ratios of sex steroids to corticosteroids) is responsible for mediating the amnestic properties of CLA requires further research. Interestingly, elevations in the ratio of cortisol to sex steroids/precursors have been reported in dementia (reviewed in [100]).

Previous studies have demonstrated CLA treatment stimulates T biosynthesis *in vitro* in a rat Leydig tumor cell line (R2C) by up-regulating CYP17A1 expression [38, 39]. However, in adult mice, gavage administration of CLA did not increase circulating T concentrations in sedentary mice, but did increase circulating T concentrations above that induced by exercise alone (6 weeks of progressive rotarod training) [39]. Similar to mice [39], in adult human males, CLA intake is linked to increased T biosynthesis following resistant exercise, but not in sedentary individuals [38]. Since we did not observe an increase in circulating T following CLA, it could be that our animals did not meet a threshold of exercise reported in other studies [38, 39]. Previous studies have shown that CLA does not alter circulating P_4_ concentrations in lactating dairy cows [101] or in luteal cells from dairy cows [102], while others have shown CLA does increase P_4_ during the luteal phase in cows [103]. CLA-induced improvements in reproductive performance of dairy cows have been attributed to CLA suppression of PGF(2α) synthesis in luteal cells and improved ovarian follicular steroidogenesis [101–103]. Interestingly, high doses of CLA (50–200 uM) but not low doses (15–25 uM), increase P_4_ production from bovine oocytes during *in vitro* maturation of parthenogenetic embryos [104]. That CLA may regulate steroidogenesis is suggested by studies demonstrating that trans-10,cis-12 conjugated linoleic acid promotes ductal elongation, since P_4_ and prolactin, independent of E_2_, are well-known to induce branching morphogenesis [61, 105].
