# Correction: Conjugated Linoleic Acid Administration Induces Amnesia in Male Sprague Dawley Rats and Exacerbates Recovery from Functional Deficits Induced by a Controlled Cortical Impact Injury

**DOI:** 10.1371/journal.pone.0188611

**Published:** 2017-11-21

**Authors:** Rastafa I. Geddes, Kentaro Hayashi, Quinn Bongers, Marlyse Wehber, Icelle M. Anderson, Alex D. Jansen, Chase Nier, Emily Fares, Gabrielle Farquhar, Amita Kapoor, Toni E. Ziegler, Sivan Vadakkadath Meethal, Ian M. Bird, Craig S. Atwood

Clarinol^®^G-80 (Lot 5385481001) was used in this study. There is an error in the description of Clarinol^®^ G-80 in the fourth sentence of the first paragraph under the "CLA Administration" subheading in the Materials and Methods section. The description should be as follows: "Clarinol^®^ G-80, is an oil mixture high in isomers of CLA (80%), comprised predominantly of c-9,t-11 (conjugated diene (CD)18:2) and t-10,c-12 (CD18:2) CLA (74.5%) with traces of oleic, palmitic and stearic acid (or safflower oil fatty acids)."

A decimal error was made in the calculation of the dosage of Clarinol^®^ intraperitoneally injected in the rats. A dose of 250mg/kg Clarinol^®^ was delivered to the rats every other day (not 25 mg/kg every other day as stated in the article). This equates to ~100 mg/kg of conjugated linoleic acid (CLA) per day and approximately two-fold the equivalent FDA recommended human dose (not half the FDA approved dose as stated in the article).

There was an error in reporting the ages of the rats used in the study. Rats were assigned to the following groups: Sham + saline group (n = 5; 5–6 months of age), Sham + CLA group (n = 7; 5–6 months of age), controlled cortical impact (CCI) injury + saline group (n = 8; 15–16 months of age), CCI injury + CLA group (n = 9; 15–16 months of age). Since the incidence of traumatic brain injury increases in the elderly, aged rats were subjected to a CCI injury. The results indicate that Clarinol^®^ at the dose injected intraperitoneally is detrimental to learning and memory in uninjured young adult rats, and limits cognitive recovery in aged rats following a CCI injury.

The conclusions of the study focus on the effects of intraperitoneal CLA administration; conclusions cannot be drawn from the study data regarding the effects of oral administration of CLA, and discussion of the effects of dietary CLA in the article is speculative. The design of our experiment related to administration of CLA was motivated by the potential use of CLA in the treatment of TBI. Many individuals with TBI, particularly those with moderate to severe TBI cannot consume food, but can take treatments i.p., s.c. or i.v. Moreover, this experimental design allows us to know the exact amount delivered to each animal and avoid complications related to differences in consumption, or differences in intestinal uptake, of CLA by different animals. Potential differences in the effects of intermittent Clarinol^®^ G-80 treatment (higher doses every other day), and between oral and intraperitoneal delivery, warrants further investigation.

To avoid the possibility of a 'control' oil inducing a physiological response that complicated the interpretation of the results, saline was administered intraperitoneally to allow similar injection/handling exposure of the animals. Various oils have previously been demonstrated to induce physiological effects.

The twelfth author's name appears incorrectly. The correct name is: Sivan Vadakkadath Meethal.
